# The Use of a Novel Porcine Derived Acellular Dermal Matrix (Mucoderm) in Peri-Implant Soft Tissue Augmentation: Preliminary Results of a Prospective Pilot Cohort Study

**DOI:** 10.1155/2018/6406051

**Published:** 2018-07-09

**Authors:** Piero Papi, Giorgio Pompa

**Affiliations:** Department of Oral and Maxillofacial Sciences, “Sapienza” University of Rome, Italy

## Abstract

**Objective:**

Over the years, several techniques have been proposed for soft tissue augmentation around dental implants in order to improve keratinized mucosa width (KMW). Recently, a porcine derived acellular dermal matrix (Mucoderm®) has been proposed as autogenous graft substitute in order to avoid palatal harvesting and obtain comparable results to connective tissue grafts, in terms of aesthetics and function. The aim of this study is to present the one-year follow-up results of this matrix in peri-implant soft tissue augmentation procedures.

**Material and Methods:**

Twelve patients were enrolled in this pilot prospective study: a dental implant was placed in the upper premolar area and, at implant uncovering after eight weeks, the matrix was inserted. KMW gain was considered as primary outcome variable.

**Results:**

After one month from matrix insertion, mean KMW was 7.86±3.22 mm (100%), with no statistically significant intragroup variations (*p*>0.05). No membrane exposures or wound healing complications occurred during postoperative phase and, after one year, mean KMW was 5.67±2.12 mm (72.13%).

**Conclusions:**

The results of the present pilot study indicate that by placing a Mucoderm membrane during implant surgery the keratinized tissue width can be augmented, and the width remains stable for the assessment period of 12 months. Further studies with greater power and longer investigation period are needed to confirm the suggestion for clinical use. Clinical trial registration number is EudraCT number 2018-000147-16.

## 1. Introduction

Dental implants are a predictable and effective treatment to replace missing teeth, with implant survival rates up to 95% after 10 years of prosthetic loading [1–3].

Implant failures can be classified as mechanical or biologic, with peri-implant diseases being considered as the most common causes of implant-related biologic complications [4–8].

Derks and Tomasi reported that 43% of dental implants were affected by mucositis and 22% by peri-implantitis after a mean follow-up of 9 years in a Swedish population [9, 10].

Peri-implantitis has been defined as chronic inflammatory lesion associated with marginal bone loss and bleeding on probing and/or suppuration, while a diagnosis of mucositis is established in case of bleeding on probing without concomitant marginal bone loss [11–16].

The role of keratinized mucosa width (KMW) around dental implants has been extensively discussed over the years: Moraschini et al. in 2017 published an overview regarding quality assessment of systematic reviews about significance of KMW for peri-implant health [17].

According to their results, questions on the ideal width of KM and its influence on implant long-term maintenance are still open.

The four studies analyzed [18–21] reported higher values for probing pocket depth (PPD), sulcus bleeding index (SbI), and plaque index (PI) in patients presenting less than 2 mm of KMW; however, data on implant survival and peri-implant diseases prevalence remained insufficient to draw clear evidence-based conclusions and lack of methodological quality was reported for all of them.

On the contrary, presence of an adequate band of KM has been correlated with better plaque control through easier oral hygiene procedures, demonstrating a positive association with healthy peri-implant soft tissues [22, 23].

Therefore, several techniques have been proposed, over the years, to increase KMW around dental implants [24–28].

Autogenous tissue grafts, including connective tissue grafts (CTG) and free gingival grafts (FGG), have shown successful results in terms of KM increasing, with FGG being considered as the gold standard [29–31].

However, tissues regenerated by FGG are characterized by differences in texture and color compared to adjacent soft tissues; therefore, its use is not recommended in aesthetic areas, where CTG are preferred.

Furthermore, either CTG or FGG require harvesting from a palatal donor site with possible postoperative morbidity and limited availability [32–34].

Recently, acellular dermal matrix has been proposed as autogenous graft substitutes in order to avoid palatal harvesting and obtain comparable results in terms of aesthetics and function [35].

A novel tridimensional porcine derived acellular dermal matrix (Mucoderm, botiss gmbh, Berlin, Germany), composed of natural types I and III collagen without any artificial cross-linking, has been developed for multiple clinical situations [35].

The aim of this study is to present the one-year follow-up results of the use of this matrix in peri-implant soft tissue augmentation procedures.

## 2. Materials and Methods

### 2.1. Study Design

To address the research purpose, the authors designed and implemented a pilot prospective cohort study to be conducted at the Department of Oral and Maxillofacial Sciences, “Sapienza” University of Rome.

Patients were recruited between subjects presenting at the university's department for dental implants placement between November 2015 and April 2016.

In order to be included in the study, patients had to meet specific inclusion criteria:

- Placement of one dental implant in the upper premolar area
- A width of the attached gingiva of less than 2 mm in the implant site
- Good oral hygiene (FMPS and FMBS < 25%)

Exclusion criteria adopted were as follows:

1. Systemic
  - Uncontrolled systemic diseases
  - Smokers (>10 cigarettes/die)
  - History of mental disorders
2. Local
  - Active periodontal disease or local inflammation
  - Previous failed implant placement or bone grafting in the site
  - Need for augmentation procedure
  - Poor oral hygiene (FMPS and FMBS > 25 %)

All patients signed the inform consent form and gave written approval to be included in the study population, according to the latest version of the World Medical Declaration of Helsinki. The institution review board of the Department of Oral and Maxillofacial Sciences, “Sapienza” University of Rome, approved the study.

### 2.2. Primary Outcome Variable

Primary outcome variable assessed was keratinized mucosa width, recorded prior to implant placement and after one, three, six, and 12 months from matrix placement.

The mucogingival line was identified by the roll test performed with a periodontal probe (UNC 15, Hu-Friedy, Chicago, IL, USA). Then, keratinized mucosa width (KMW) was measured with a rotating movement of the probe by placing the tip at the mucogingival junction and continuously adapting the probe's axis on the curved surface of the gingiva up to the zenith of the alveolar ridge. After prostheses delivery, crowns were taken off in order to allow the assessment. The measurement was performed by the same examiner (PP) at all interval of times considered.

Furthermore, a photograph of the edentulous area was taken vertically to the occlusal plane in the central position of the missing tooth. A digital camera (Nikon D7100, Nikon, Japan) was used with standardized settings (ISO 200, F32, shutter speed of 1/160) preoperatively and at all follow-up visits. The image was saved on a personal computer (Macbook Pro, Apple, CA, USA) and KMW was calculated through a professional photo editing software (Photoline 20, Computerinsel GmbH, Germany).

### 2.3. Secondary Variables

#### 2.3.1. Marginal Bone Level

Mesial and distal implant crestal bone levels were measured on standardized periapical radiographs (Rinn, York, PA, USA). The radiographs were evaluated by an independent investigator and expert in the field.

The reference point for the bone level measurement was the implant shoulder. The bone level was evaluated by measuring the distance between the implant shoulder and the first visible bone contact on the implant. The bone level measurements were recorded on the mesial and distal aspect of each implant.

#### 2.3.2. Implant Survival

An implant in place at the respective follow-up visit was considered as surviving implant.

#### 2.3.3. Implant Success

Implant success was documented according to the following criteria defined by Buser et al. (1990) [36]:

- Absence of persistent subjective complaints, such as pain, foreign body sensation, and/ or dysaesthesia
- Absence of a recurrent peri-implant infection with suppuration
- Absence of mobility
- Absence of a continuous radiolucency around the implant

#### 2.3.4. Soft Tissue Assessment (mSBI, PI, and PPD)

*Plaque Index*. The Plaque Index (PI) was determined on the mesial, buccal, distal, and palatal surfaces of the implant, according to Mombelli et al. (1987) [37]:

- Score 0: no plaque detected
- Score 1: plaque only recognized by running a probe across the smooth marginal surface of the implant
- Score 2: plaque can be seen by the naked eye
- Score 3: abundance of soft matter

*Sulcus Bleeding Index*. Determined on the mesial, buccal, distal, and palatal surfaces of the implant according to Mombelli et al. (1987) [37]:

- Score 0: no bleeding when a periodontal probe is passed along the gingival margin adjacent to the implant
- Score 1: isolated bleeding spot visible
- Score 2: blood forms a confluent red line on margin
- Score 3: heavy or profuse bleeding

*Probing Pocket Depth*. The probing pocket depth (PPD), expressed in millimeters (mm), is the distance from the gingival margin to the bottom of the probable pocket at 4 sites (mesial, buccal, distal, and palatal) of the implant.

### 2.4. Surgical Procedure

One hour prior to surgery, prophylactic antibiotics were given to patients: 2 gr of amoxicillin and clavulanic acid (Augmentin®, Roche S.p.A., Milan, Italy) or, in case of allergy, 500 mg of azithromycin (Zitromax, Pfizer, New York, USA).

A mucoperiosteal flap was raised in the edentulous ridge of the premolar area and intrasulcular incisions were performed in the adjacent teeth, using a 15c scalpel blade (Hu-Friedy, Chicago, IL, USA).

**Figure 1:**
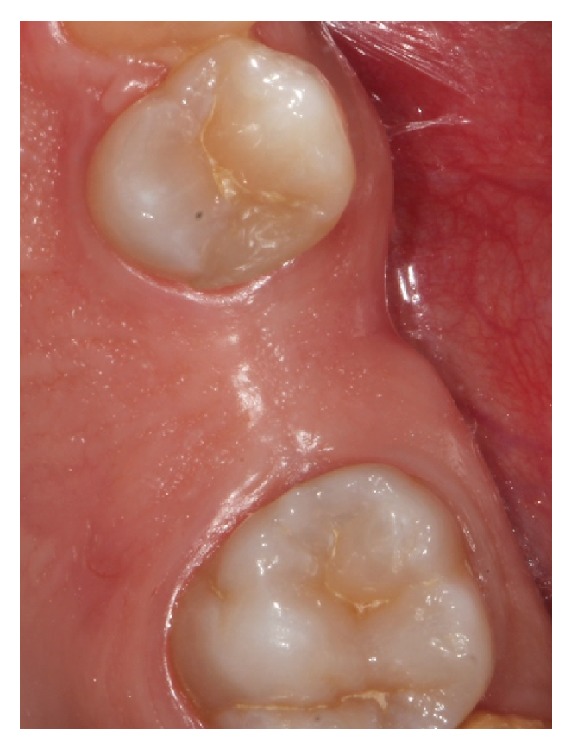
Preoperative clinical picture.

**Figure 2:**
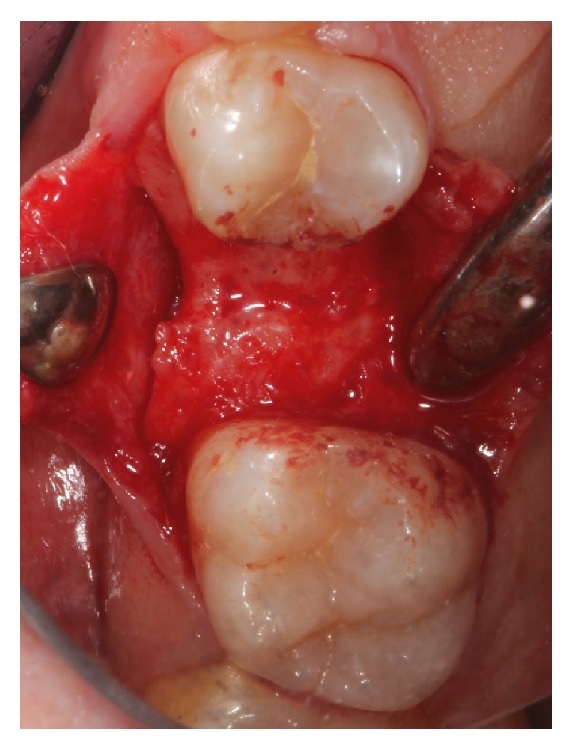
Preoperative situation after full thickness flap elevation.

**Figure 3:**
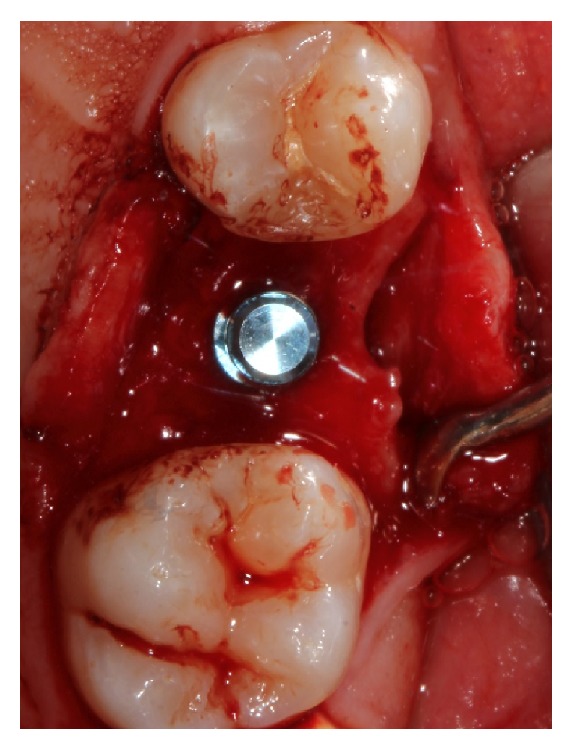
Implant in place.

A titanium-zirconium dental implant was placed following proper manufacturer's instructions (Bone Level Tapered Roxolid, Institut Straumann AG, Basel, Switzerland) with adoption of a submerged healing protocol; sutures (Vycril 4.0, Ethicon, Johnson and Johnson, New Brunswick, NJ, USA) were removed after 10 days (Figures 1–3).

Medical check-outs were, then, scheduled every 15 days.

**Figure 4:**
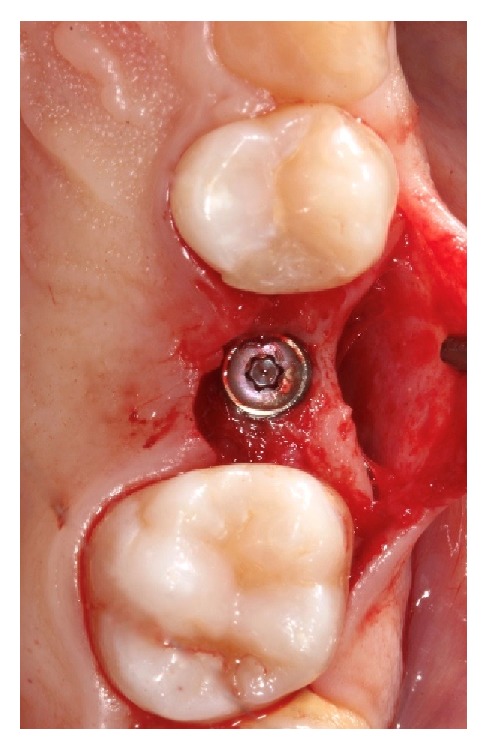
Implant uncovering after eight weeks.

**Figure 5:**
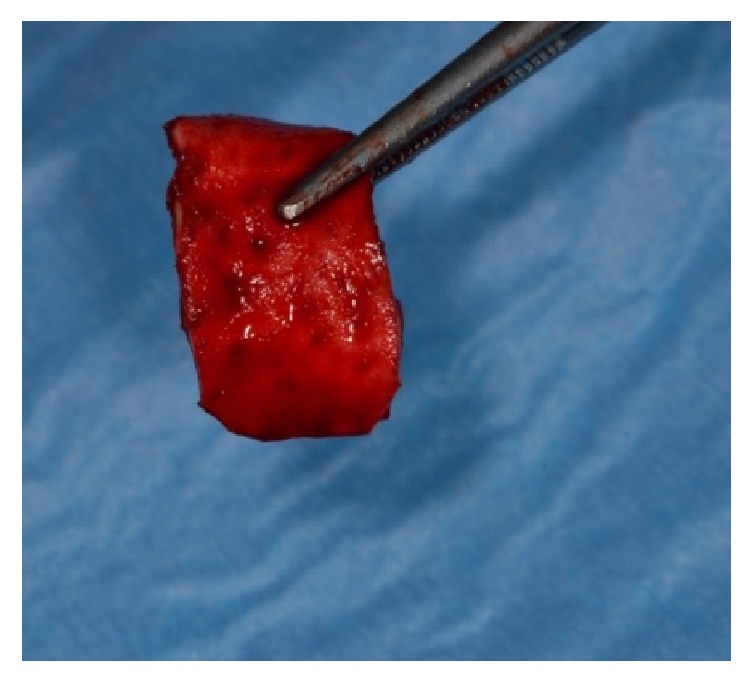
Matrix hydrated in fresh human blood.

**Figure 6:**
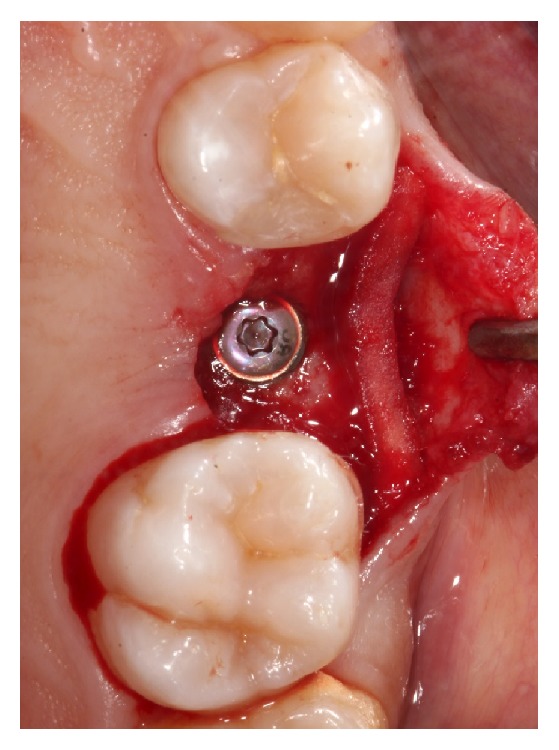
Matrix placement.

A second surgery for implant uncovering was performed after 8 weeks, with an early loading protocol adopted [38], as defined by the 5th ITI Consensus Conference [39]: a palatal u-shape incision was designed to preserve keratinized gingiva and a split-thickness flap was elevated vestibulary. A subepithelial pouch was prepared and a PDCM matrix (Mucoderm, botiss gmbh, Berlin, Germany) of 15 mm of height and 20 mm of length was adapted based on patient's need and passively fitted. The matrix was sutured to the corresponding periosteum with interrupted absorbable sutures (Vycril 6.0, Ethicon, Johnson and Johnson, New Brunswick, NJ, USA) (Figures 4–6).

Before placement, matrix was hydrated for 10 minutes in fresh human blood collected after flap elevation in each patient.

Healing collars were inserted and flaps were sutured (Vycril 4.0, Ethicon, Johnson and Johnson, New Brunswick, NJ, USA).

Patients were instructed to rinse twice a day with an antiseptic mouthwash with chlorhexidine 0.2% (Curasept, Curaden Healthcare S.p.A, Saronno, Italy) for 60 seconds starting for 10 days; a soft diet was recommended and ibuprofen 600 mg (Brufen, Abbott, Verona, Italy) was prescribed to be taken as needed.

After suture removal, impressions to create an individual impression tray were obtained. After 21 days of healing, new definitive impressions with polyether impression material (Impregum, 3M ESPE AG) were taken with an open-tray using suitable impression copings to deliver provisional PMMA restorations.

**Figure 7:**
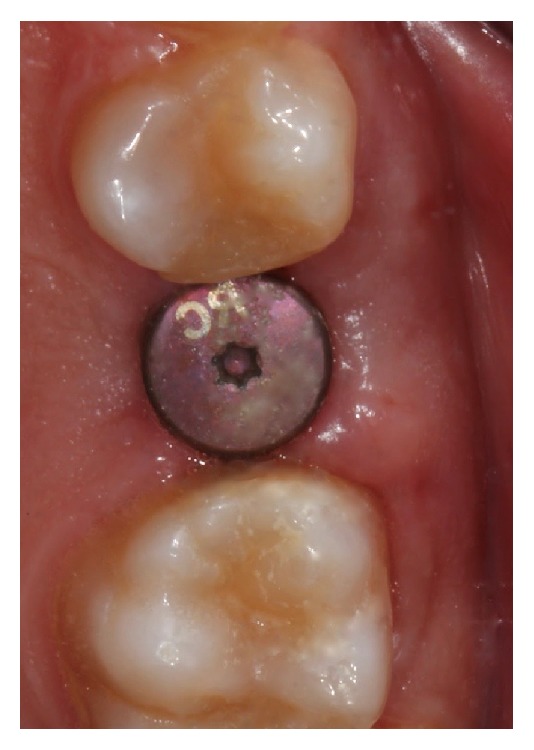
15 days postoperative examination.

**Figure 8:**
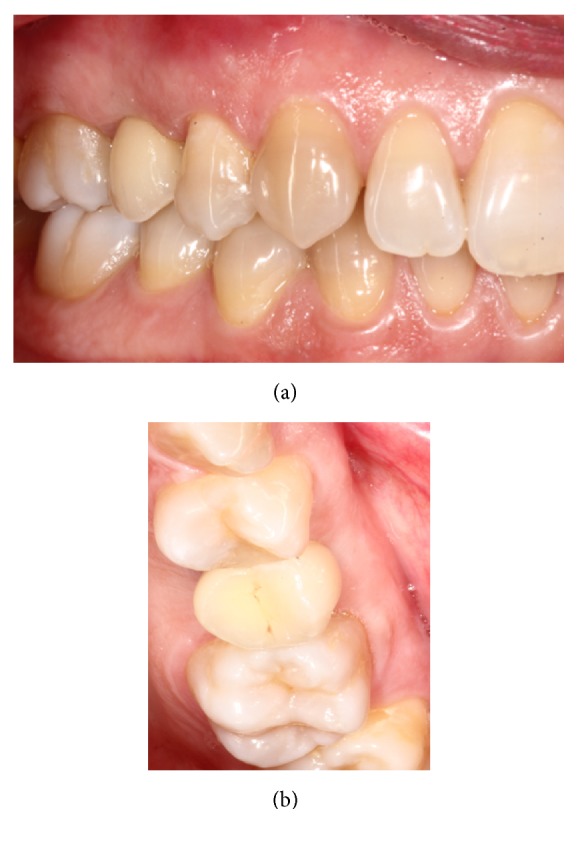
(a)-(b) 1-year postoperative examination.

Two months later, definitive gold-ceramic prostheses were inserted (Figures 7 and 8). In case of iso- or supramucosal preparation borders the crowns were cemented; in situations with submucosal preparation the crowns were screw retained at 30 Ncm.

Follow-up visits were scheduled once a month; primary and secondary outcome variables were assessed at baseline (secondary surgery) and at three, six, and twelve months.

### 2.5. Statistical Analysis

Descriptive statistics were calculated (mean, range, and standard deviations) for each variable of the study. Mean values of KMW expressed in millimeters were calculated for each patient at each examination time point and additionally expressed as percentages relative to the immediate postoperative (day 0) measurement (defined as 100%). For intragroup comparison, the nonparametric Mann–Whitney U-test was used with a p value <0.05 considered as statistically significant.

Specific statistical software (IBM SPSS V10 Statistics, IBM, Armonk, USA) was used to analyze the data.

## 3. Results

A total of twelve patients were enrolled in this study; they were either males (5) or females (7), with a mean age of 43.75 ± 7.97 years (range= 32-56 years).

Keratinized mucosa width mean value recorded prior to implant treatment was 1.35±0.32 mm.

One dental implant (Bone level tapered, Institut Straumann AG, Basel, Switzerland) was placed in the premolar edentulous area of each patient, according to bone availability and following manufacturer's instructions.

Sample and implant characteristics are described in Table 1.

All implants were uncovered after eight weeks; no adverse reactions or events were reported in the postoperative phase. Primary wound closure was obtained in all cases.

After one month from PDCM insertion, mean keratinized mucosa width was 7.86±3.22 mm, with no statistically significant intragroup differences (*p*>0.05).

No membrane exposures or wound healing complications occurred during postoperative phase.

After one year, mean KMW was 5.67±2.12 mm (72.13%), with no statistically significant intragroup variations (*p*>0.05); all values of KMW are reported in Table 2.

As for secondary outcome variables, no implant was lost at follow-up and implant success and survival rates were 100%. No PPD values >5 mm were registered, with no concomitant signs of inflammation. Bleeding on probing occurred just in one patient after 6 months (*p*>0.05): implant crown was professionally cleaned with polishing paste and rubber cup, patient was prescribed to rinse twice a day with an antiseptic mouthwash with chlorhexidine 0.12% for 10 days, and strict oral hygiene instructions were provided.

At following appointments, no bleeding on probing was detected.

Average marginal bone loss was 0.38±0.21 mm after 1 year of prosthetic loading (Table 3).

## 4. Discussion

Influence of KM width on peri-implant health is still controversial and not adequately supported in literature; however, based on clinical experience, several authors have showed that soft tissue thickening procedures around dental implants are generally associated with an improving in aesthetics, function, and lower complications rates [40–42].

A novel porcine derived acellular dermal matrix has been recently introduced to avoid donor site harvesting: Pabst et al. demonstrated, in an vitro study, a significant integration with surrounding tissues and its excellent revascularization properties [43].

Park et al. investigated cell proliferation characteristics of the novel PDCM: host cell migration and penetration in the tridimensional architecture of the matrix is enhanced by its interconnected structure, allowing microvessels formation and neoangiogenesis [44].

They reported how adding enamel matrix derivative (EMD) or platelet-rich fibrin (PRF) may increase these properties, improving vascularization around and through the collagen matrix.

Immune response induced by collagen membranes has been extensively investigated [45, 46]: cross-linking agents have been used in order to prolong the degradation period and to improve mechanical properties, with glutaraldehyde as mostly used.

However, these agents may produce cytotoxic effects and show a limited proregenerative macrophage recruitment [47, 48].

The novel PDCM has no special treatment to augment cross-linked fibers to avoid immunogenic reactions [44].

Rothamel et al. evaluated, in an in vivo animal study in rat model, biodegradation pattern and stability of PDCM, concluding that substitution of the dermal matrix with newly formed collagen tissue occurred in around six to nine months [35].

Different rehydration protocols have been proposed, depending on its clinical application and flexibility required: Kasaj et al. evaluated changing in biomechanical properties and concluded that there was a strong indication to immerse the matrix into sterile saline solution or fresh human blood for around 10 minutes, with the latest to be preferred in order to enhance tissue integration properties [49].

According to the latest American Academy of Periodontology Regeneration Workshop and the 4^th^ European Academy of Osseointegration consensus conference, studies on dermal substitutes have shown sufficient evidence to support their usage in soft tissue augmentation and thickening procedures around teeth and dental implants [50, 51].

Sanz et al. [52] compared in a randomized clinical trial the benefits of an acellular dermal matrix with the application of a connective tissue graft in KM augmentation around teeth with fixed prosthetic restorations. According to their results, the test group showed a mean KM gain of 2.5 mm compared with 2.6 mm of the control group (CTG), with both groups showing a contraction of KMW during the study period of 3 months.

Lorenzo et al. [53] utilized a PDCM for soft tissue peri-implant augmentation, obtaining a 2.9 mm gain of KMW after 6 months that was comparable to the 2.8 mm gain of the control group (CTG), with a mean shrinkage of the matrix amounting for around 60% of the original dimension.

In 2015, Vignoletti et al. [54] histologically evaluated the healing of the PDCM in KM augmentation around teeth in an animal study.

According to their results, the matrix was no longer identifiable at three months after insertion and demonstrated full integration in the surrounding connective tissues.

Schmitt et al. investigated the use of a PDCM in vestibuloplasty procedures around dental implants in mandibular overdentures [26]: they reported the longest follow-up available in literature, with data up to five years, comparing free gingival grafts with PDCM for KMW augmentation.

The two groups showed comparable results in terms of KMW gain (8.4 mm versus 6.15 mm, respectively), with color appearance and esthetic results of PDCM group significantly better compared to FGG group.

However, the PDCM group showed a final shrinkage of 52.9 % from baseline compared to 40.7 % of the FGG group.

Thoma et al. [55], in 2018, performed a systematic review with meta-analysis, evaluating soft tissue augmentation or thickening procedures around dental implants. They included 10 studies and concluded that the apically positioned flap (APF) in conjunction with autogenous grafts resulted in a pronounced improvement of peri-implant health.

Vignoletti et al. [56] reported a 4.5 mm mean gain of KMW for APF plus autogenous grafts (either FGG or CTG) in the 12 studies included in their narrative review.

Several surgical techniques can be used for KMW augmentation or soft tissue thickening at second-stage surgery [57]: However, it is clinically important to know that with APF, roll envelope flap, and even split-thickness skin graft a certain postoperative shrinkage has to be expected for all grafting procedures.

To the best of the authors' knowledge, this is one of only few studies [58, 59] to test in vivo this novel PDCM for soft tissue augmentation procedures around dental implants.

According to our results, a stable and effective gain of KMW can be obtained, avoiding limited availability and postoperative morbidity of having a donor site.

Key is stabilization of the matrix that can be easily achieved by suturing it to the periosteum, once the membrane is sufficiently rehydrated in fresh human blood derived from patient.

Main limitations are represented by the small sample and absence of a control group: this pilot prospective cohort study was designed by the authors to obtain sufficient data on expected KMW gain, to conduct and implement a randomized control clinical trial, comparing the novel PDCM to connective tissue grafts in soft tissue augmentation procedures around dental implants, with an appropriate follow-up (five years) and a larger sample.

The KMW gain was associated, also, with implant uncovering technique and the u-shape palatal incision: a shrinkage was experienced at all intervals measured, in accordance with values reported in literature using either autogenous grafts or other PDCM matrix [26, 53–57].

## 5. Conclusions

Our results indicate that by placing a Mucoderm membrane at implant second-stage surgery, in case of soft tissue deficiencies, the keratinized tissue width was augmented, with shrinkage consistent with previous publications. Further studies with greater power and longer investigation period are needed, with a proper randomized control clinical trial to confirm the suggestion for clinical use.

## Figures and Tables

**Table 1:** Sample demographics.

Study variable	Descriptive statistics

Sample size (n)	12

Male	5

Female	7

Age (y) ± SD (range)	43.75 ± 7.97 years (range= 32-56 years).

Dental Implants Bone Level Tapered	

Diameter 4.1 mm	8

Diameter 3.3 mm	4

Length 8 mm	2

Length 10 mm	6

Length 12 mm	4

**Table 2:** Keratinized mucosa width (KMW) was measured with a periodontal probe (UNC 15, Hu-Friedy, Chicago, IL, USA) from the zenith of the alveolar ridge to the mucogingival junction and expressed in mm and %. Baseline is defined as secondary surgery and implant uncovering.

Examination Time Point	Mean KMW (mm)	Mean KMW (%)	Intragroup comparison ( *p <0.05)*
Pre-Operative	1.35±0.32	-	

1 month	7.86±3.22	100	*p>0.05*

3 months	7.03±1.23	89.44	*p>0.05*

6 months	6.43±1.89	81.80	*p>0.05*

12 months	5.67±2.12	72.13	*p>0.05*

**Table 3:** Secondary outcome variables. PPD= Probing pocket depth, SbI= Sulcus Bleeding Index, PI= Plaque Index, N/A= Not Assessed.

Examination Time Point	Marginal Bone Loss (mm)	Implant survival (%)	Implant success (%)	PPD (mm)	SbI	PI	Intragroup comparison ( *p <0.05)*
Baseline	0.17 ± 0.25	100	100	N/A	N/A	N/A	*p>0.05*

3 months	0.24±0.08	100	100	3.5	0.08	0.23	*p>0.05*

6 months	0.29±0.12	100	100	3.75	0.25	0.41	*p>0.05*

12 months	0.38±0.21	100	100	3.5	0.08	0.75	*p>0.05*

## Data Availability

The datasets used to support this study are currently under embargo, while the research findings are commercialized. Requests for data, 12 months after initial publication, will be considered by the corresponding author.
